# Hydrothermal *in situ* synthesis of Rb and S co-doped Ti-based TiO_2_ sheet with a thin film showing high photocatalytic activities†

**DOI:** 10.1039/c7ra12757j

**Published:** 2018-03-21

**Authors:** Yuhan Wang, Chunli Kang, Dongmei Huang, Kunkun Xiao, Ling Zhu, Fang Liu, Tao Tian

**Affiliations:** Key Laboratory of Groundwater Resources and Environment, Jilin University, Ministry of Education Changchun 130021 Jilin P. R. China tiantao@jlu.edu.cn +86 13843132281

## Abstract

TiO_2_ is considered as one of the most promising semiconductor photocatalysts used to degrade organic pollutants. Element doping has a good effect on improving the properties of TiO_2_. Herein, by using Rb_2_SO_4_, we explored the *in situ* synthesis of Ti-based TiO_2_ sheets with a thin film through a hydrothermal reaction. Then, the photocatalyst was successfully fabricated by calcination. All samples were characterized by FT-IR, XRD, SEM, XPS, PL and UV-vis DRS measurements. The results indicate that the S doping together with surface hydroxyl groups lead to the band gap narrowing. S and a trace amount of Rb element can enable the formation of uniform microspheres on the surface of the Ti plate and the major phase transformed from titanium to anatase. The band gap absorption extended from 400 nm to 600 nm. The photocatalytic properties were investigated by performing the degradation of methyl orange (MO) and 4-chlorophenol (4-CP) in the aqueous solutions under UV and simulated sunlight. In the series of TiO_2_ photocatalysts, Rb/S/TiO_2_-48 shows the best photocatalytic efficiency and good photocatalytic performance on recycling. Interestingly, when H_2_O_2_ was added to the MO aqueous solution, a synergistic effect of the TiO_2_ thin film and H_2_O_2_ on degrading the pollutant was observed.

## Abstract 2

Rb_2_SO_4_ helps the *in situ* synthesis of the TiO_2_ thin film with microsphere and needle-like structure, which shows the remarkable photocatalytic properties.

## Graphical abstract

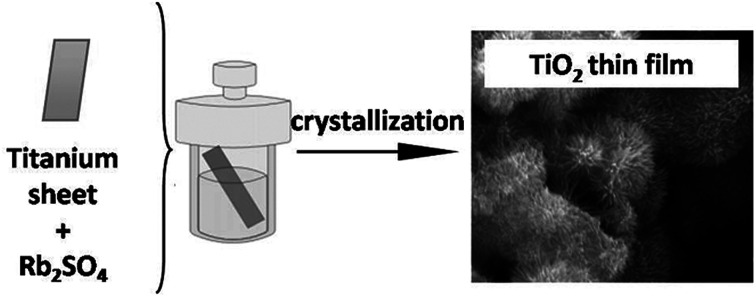

## Introduction

In recent years, with the development of industrial society, organic wastewater has become a serious problem and thus, the improvement in sewage disposal technologies has drawn significant attention. Among many methods, photocatalysis, which utilizes solar energy, is labeled as a promising green chemistry technology.^1,2^

Titanium dioxide (TiO_2_) is a type of semiconductor material, which has aroused wide research interest because of its non-toxicity, low cost, excellent thermal and chemical stability, strong optical absorption, and great electronic and catalytic properties.^3–5^ It is one of the most familiar semiconductor photocatalysts for the degradation of organic pollutants,^6–8^ the manufacture of dye-sensitized solar cells,^9^ the photocatalytic hydrogen generation,^10,11^ and so on. However, due to the large band gap of TiO_2_ (3.2 eV),^12,13^ only when the wavelengths of the light are less than 400 nm, the electron–hole pairs would appear. Moreover, only UV light and 5% of the solar spectrum can be utilized,^14^ resulting in a low efficiency of the utilization of sunlight. The easy recombination of the light-generated electrons and holes also limits the practical application of TiO_2_.^15^ Thus, it is essential to enhance the efficiency of this material. Many modification strategies have been demonstrated to improve the photocatalytic efficiency of TiO_2_, which can shift the absorption wavelength towards the visible light and extend the existence time of the electron–hole pairs. TiO_2_ has been doped with metals,^16–18^ anions,^19–22^ modified by dye sensitization^23–25^ and surface modification,^26,27^ coupled with other semiconductors,^28–30^ and so on. These methods transform the lattice type of TiO_2_, improve its performance and modify the structure of its sol or film surface. This leads to introduction of surface defects and broadening the absorption range of TiO_2_ in the visible light region.

Among numerous methods, an *in situ* synthesis method to prepare a TiO_2_ film is considered as an effective way to improve the properties of TiO_2_.^31,32^ This method has shown some advantages such as excellent adhesion between TiO_2_ film and the matrix, a simple operation procedure, and good control of the particle size and the crystalline phase. In addition, this method also helps to overcome the shortcomings of a powder catalyst, such as easy agglomeration or the requirement of a post-filtration procedure for recovering the catalyst from treated sewage water.^33,34^ In this process, a titanium sheet is used to form the TiO_2_ film on the original titanium sheet surface. As a new green preparation method, metallic titanium is the source of titanium rather than titanium alkoxides; other materials are also inorganic substances, which reduce the quantity of contaminants generated during the preparation process. The TiO_2_ film has one- or multi-dimensional structure with special morphology, which grows directly out of the surface of TiO_2_, resulting in the good adhesion with the matrix. The film also shows a stable performance caused by the quantum size effect, the large surface area, and the surface and interface effects of different TiO_2_ structures. In a previous study, Wang *et al.* synthesized a 2-dimensional TiO_2_ nanofilm *in situ* by a hydrothermal method. Its crystalline structure was a mix-crystal structure of anatase and TiO_2_ (B), which showed a better performance in the degradation of Rhodamine B when compared with a benchmark P25.^35^ Zhang *et al.* fabricated single-crystal TiO_2_ nanorod arrays by the *in situ* growth on a Ti substrate with the help of anodic aluminum oxide (AAO). The well-aligned TiO_2_ NR arrays showed good single crystallinity after annealing, which was an advantageous for the potential applications in photocatalysis.^36^ Jiang *et al.* prepared TiO_2_-graphene composites by the *in situ* growth of TiO_2_ at the interlayer of inexpensive expanded graphite (EG) under solvothermal conditions. These composites showed high photocatalytic activity in the degradation of phenol under visible and UV lights in comparison with that using bare Degussa P25.^37^

Herein, a new Rb_2_SO_4_-modified TiO_2_ thin film with needle-like structure growing on the microspheres was synthesized *in situ*, followed by calcination. This photocatalyst could improve the efficiency of solar energy utilization by extending the absorption wavelength range, making it possible to capture the UV and visible light. The new features of the TiO_2_ film on the sheet enable the acceleration of the charge and energy transfers of TiO_2_, which was realized by the promotion of the photocatalytic activity. Furthermore, the photocatalytic performance of the TiO_2_ films prepared with different times was investigated by performing the degradation reaction of methyl orange (MO) and 4-chlorophenol (4-CP). In addition, the synergistic effect of H_2_O_2_ and the Rb and S co-doped TiO_2_ films on decolorization of MO was also studied in detail.

## Experimental

### Materials and methods

All reagents used in this study were of analytical grade and used without further purification. The material composition and chemical bonding were examined by recording the Fourier-transform infrared spectra (FT-IR, IFS 66V/S, Germany). Phase identification of the TiO_2_ films was carried out using powder X-ray diffraction patterns collected on a Rigaku Ultima IV system with the diffraction angle range from 10° to 80°. The morphology of the as-prepared TiO_2_ films was studied by scanning electron microscopy (SEM, JSM-6510). UV-vis diffuse reflectance spectra of all the samples were obtained on a U-4100 UV-vis spectrophotometer. The elemental analysis and chemical states of different atoms were examined through X-ray photoelectron spectroscopy (XPS, ESCALAB 250). The photoluminescence (PL) emission and excitation (PLE) properties of the prepared materials were studied using an F-7000 photoluminescence spectrometer (Hitachi, Japan). The photodegraded samples of MO and 4-CP were analyzed using a UV-vis spectrophotometer (UVmini-1240, SHIMADZU) and high-performance liquid chromatography (HPLC, LC-20, SHIMADZU), respectively.

### Synthesis of photocatalyst

**Fig. 1:**
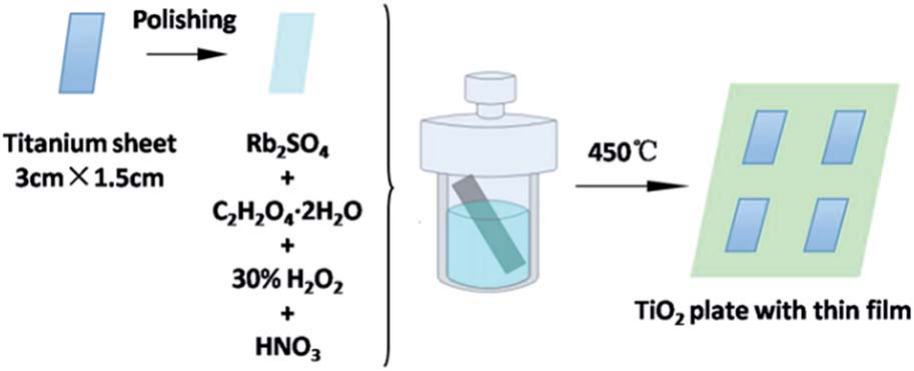
Illustration of the synthesis of the Rb_2_SO_4_-modified TiO_2_ sheet with a thin film for different preparation times.

The Rb_2_SO_4_-modified TiO_2_ sheet with a thin film was prepared *in situ* through a hydrothermal method. In a typical procedure, the titanium sheets were polished using sandpapers with the size of 600Cw, 800Cw, 1000Cw, and 1200Cw. The polished sheets were cut to the uniform specification (3 cm × 1.5 cm). A polishing solution (1 mL HF and 10 mL HNO_3_) was used to perform further chemical polishing for 60 s. Following this, the sheets were sonicated with deionized water and dried under naturally. Then, the sheets were placed in 20 mL autoclaves, which contained Rb_2_SO_4_ (0.16 g), C_2_H_2_O_4_·2H_2_O (1.248 g) and 0.2 M HNO_3_ solution containing 20 mL H_2_O_2_ as a solvent. The autoclaves were heated to 80 °C and kept for different durations (*i.e.*, 24 h, 30 h, 48 h, and 72 h). Finally, the sheets were calcined in a muffle furnace at 450 °C for 1 h; the obtained products were designated as Rb/S/TiO_2_-24, Rb/S/TiO_2_-30, Rb/S/TiO_2_-48 and Rb/S/TiO_2_-72, respectively. For comparison, the TiO_2_ sheet without Rb_2_SO_4_ was also prepared using the same method and was designated as TiO_2_-undoped (*i.e.*, the Ti plate fabricated without Rb_2_SO_4_) (Fig. 1).

### Photocatalytic activities

The photocatalytic activities of the samples were evaluated by monitoring the photodegradation of 5 mg L^−1^ MO solution and 10 ppm 4-CP solution using an 80 W high-pressure mercury lamp and a 500 W Xe lamp as the light sources, respectively. The irradiated aqueous solution contained a slice of the as-prepared TiO_2_ sheet. Before irradiation, the solution was magnetically stirred in the dark for 30 min to attain the adsorption–desorption equilibrium. Then, the solution was irradiated under magnetic stirring for the entire duration of the reaction. For monitoring the photodegradation, 3 mL of the solution was withdrawn at regular time intervals and centrifuged. The MO samples were analyzed by recording the largest absorption peak at 464 nm using a UV-vis spectrophotometer. The 4-CP samples were evaluated by HPLC. The recycling tests were also performed according to the abovementioned procedure. On the basis of the above experiments, 0.01 mL H_2_O_2_ was added to the system to study the synergistic effect of different TiO_2_ films and H_2_O_2_ on the degradation of the contaminants. Finally, the function of different reactive species on the pollutant removal was clarified by a scavenger study.

## Results and discussion

**Fig. 2:**
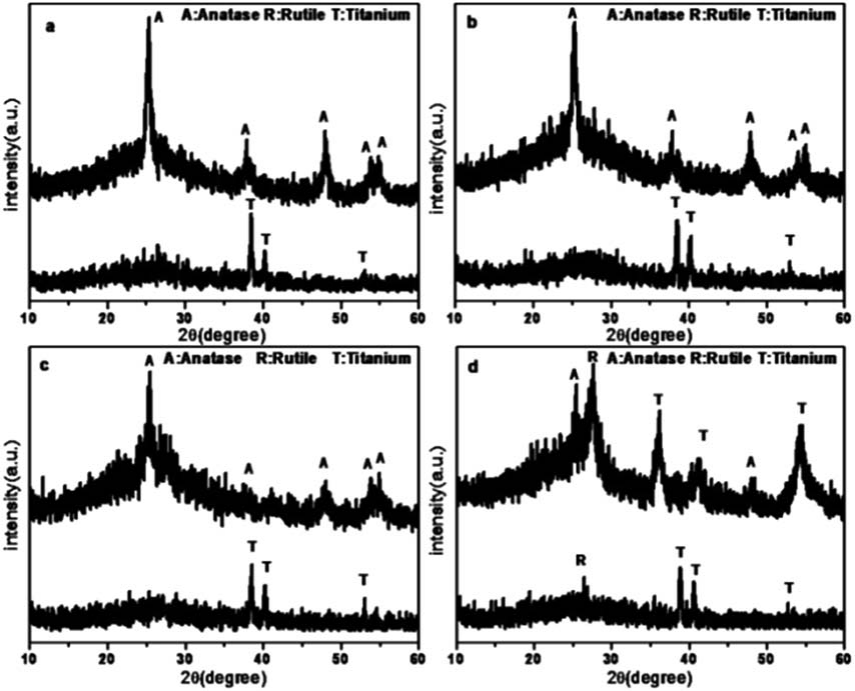
XRD patterns of the Rb and S co-doped TiO_2_ films with different preparation times: (a) Rb/S/TiO_2_-24, (b) Rb/S/TiO_2_-30, (c) Rb/S/TiO_2_-48, and (d) Rb/S/TiO_2_-72.

The XRD patterns of the TiO_2_ films are shown in Fig. 2. As shown in the patterns, the main peaks of the anatase phase are located at 2*θ* = 25.325°, 37.813°, 47.981°, 53.942°, and 54.996°, which are corresponding to the lattice planes of (101), (004), (200), (105), and (211), respectively. The peaks of the Ti phase are located at 2*θ* = 38.513°, 40.148°, and 53.006°. According to the XRD patterns, the TiO_2_ film before calcination contained rutile and titanium. In contrast, after calcination, these two phases disappeared and new peaks were found. The dominant phase of the TiO_2_ film has changed significantly. After calcination at 450 °C for 1 h, the major phase was transformed from titanium to anatase, which has been widely accepted to be efficient in pollutant removal.^38,39^ However, in the case of Rb/S/TiO_2_-72, the major phase was still titanium.

**Fig. 3:**
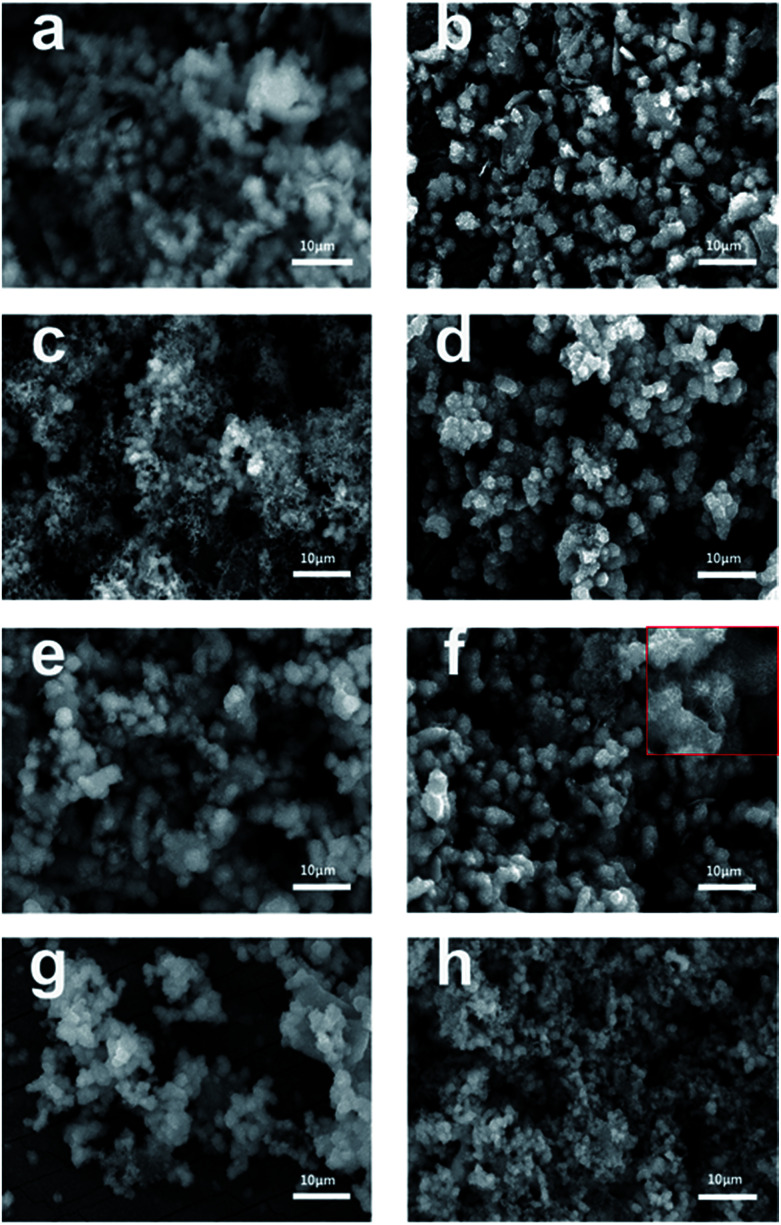
SEM images of the Rb and S co-doped TiO_2_ films with different preparation times: (a, b) Rb/S/TiO_2_-24, (c, d) Rb/S/TiO_2_-30, (e, f) Rb/S/TiO_2_-48, and (g, h) Rb/S/TiO_2_-72.

The morphology of the synthesized TiO_2_ films before and after calcination was characterized by scanning electron microscopy (SEM). As shown in Fig. 3a–f, uniform microspheres were obtained through a one-step hydrothermal reaction and the average diameter of the microspheres was about 3 μm. After calcination, as shown in Fig. 3b, d and f, the microspheres with a rougher surface became increasingly clear. However, for Rb/S/TiO_2_-72 (Fig. 3g and h), the film with large area peeled off and the diameter of the microspheres was much smaller, leaving the substrate exposed and resulting in a reduction in the surface area of Rb/S/TiO_2_-72. The XRD patterns reveal that the anatase phase is the major component of the microspheres. As shown in SEM images, the microsphere structures in the calcined TiO_2_ films with different preparation times have similar size and density. All the characterizations proved that after calcination, the TiO_2_ thin films were obtained *in situ*. When the magnification was increased (the inset in Fig. 2f), a good distribution of the microspheres in the former three samples can be clearly observed. Moreover, there are uniform needle-like structures observed on the surface of each microsphere (particularly in Rb/S/TiO_2_-48). It is reported that together with the S element, a certain amount of the Rb element on the film can enable stable adhesion with a complete coverage on the original Ti plate and induce the formation of a special morphology.^40,41^ Photons can be captured effectively due to the small size and large surface area and in turn, the large surface area will provide more active sites, which establishes full contact between the contaminant molecules and the TiO_2_ film, leading to an enhancement in the photocatalytic performance. However, in Rb/S/TiO_2_-72, the microspheres collapsed and the needle-like structure disappeared, resulting in an uneven morphology.

**Fig. 4:**
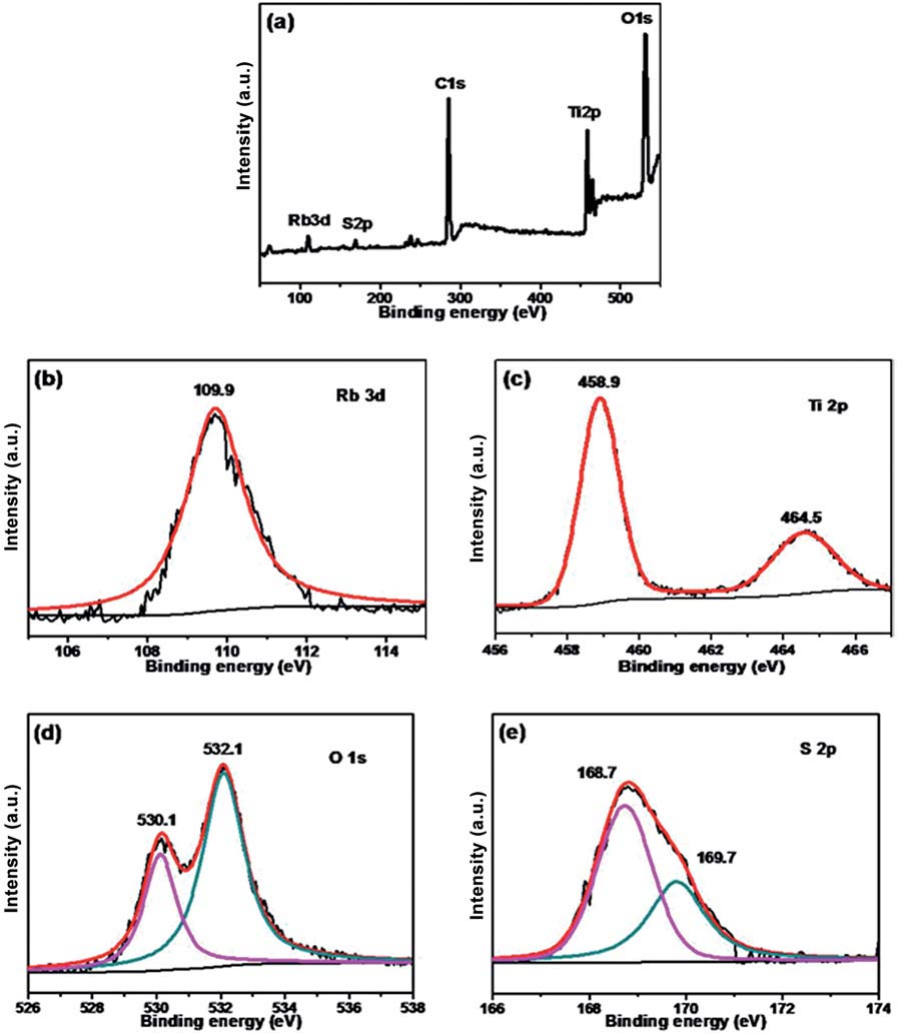
XPS spectra of the Rb/S/TiO_2_-48 film: (a) full scan XPS spectrum, (b) Rb 3d, (c) Ti 2p, (d) O 1s, and (e) S 2p.

The chemical states of different atoms were examined by X-ray photoelectron spectroscopy (XPS). As shown in Fig. 4, taking Rb/S/TiO_2_-48 as an example, the photocatalyst contains Rb, Ti, O, C and S elements. The C 1s peak at 284.6 eV can be ascribed to the adventitious carbon. The Rb 3d peak is located at 109.9 eV, which confirms the existence of the Rb element in the film. In Fig. 4c, two major emission peaks are observed at 458.9 eV and 464.5 eV for Ti 2p_3/2_ and Ti 2p_1/2_, respectively, indicating the existence of titanium in the Ti^4+^ state in the tetragonal structure of anatase TiO_2_. In Fig. 4d, the strongest peak at 532.1 eV was the result of the presence of hydroxide absorbed on the Rb and S co-doped TiO_2_ film. Another peak at 530.1 eV was related to the lattice oxygen in the crystalline TiO_2_.^42^ The S 2p core level energy spectrum shows a broad peak, which arises due to the overlap of the split sublevels (2p_3/2_ and 2p_1/2_) by spin–orbit coupling. The peak observed at 168.7 eV and 169.7 eV are attributed to 2p_3/2_ and 2p_1/2_, respectively, which is the evidence of the existence of S^6+^ in the form of chemisorbed SO_4_^2−^ species in the lattice. Due to the substitution of Ti atom by S atom, the linkage might be similar to Ti–O–S.^43^ Combining these results with the FT-IR results (Fig. S2†), it can be concluded that the peaks at 932–1230 cm^−1^ are characteristic of the chemisorbed sulphate groups, which disappear only in the case of Rb/S/TiO_2_-72.

**Fig. 5:**
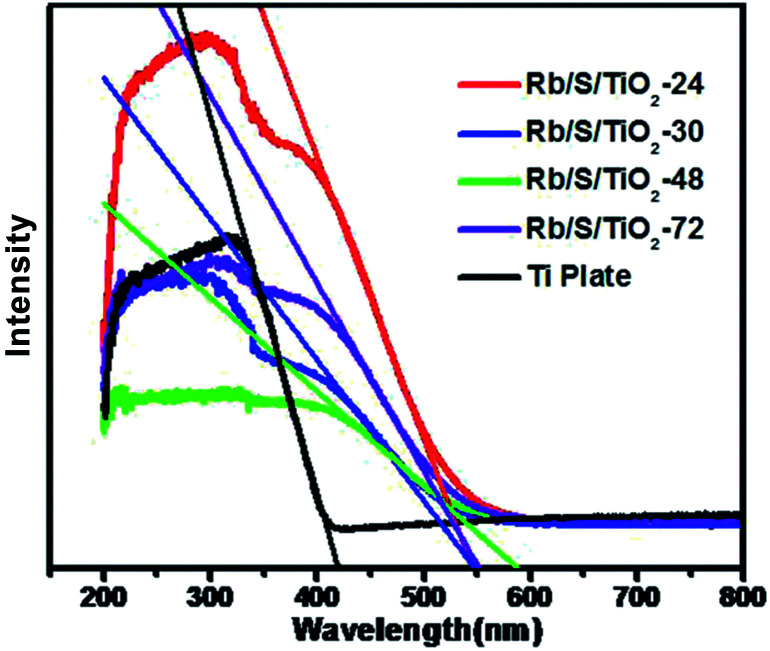
UV-vis DRS spectra of the Rb and S co-doped TiO_2_ films and pure Ti plate.

As shown in Fig. 5, the UV-vis DRS spectra of the TiO_2_ films with different preparation times were analyzed in comparison with the untreated Ti plate. The analysis was performed in the wavelength range of 200–800 nm. It can be seen that the absorption range of the pure Ti substrate is shorter than 400 nm. This absorption range is similar to that of pure TiO_2_, which is mainly due to the band–band electronic transitions of TiO_2_ (O^2p^ → Ti^3d^). Compared to the untreated Ti plate, the TiO_2_ films exhibited enhanced absorption in the visible-light region. The absorption maximum of the TiO_2_ films was at around 600 nm, which indicates the broadening of the absorption range in comparison to pure TiO_2_. The doped S element results in the generation of an impurity energy level between the valence band (VB) and the conduction band (CB) in the lattice of TiO_2_, which leads to the narrowing of the band gap. Based on the Kubelka–Munk function (eqn (1))

1 *E*_g_ = 1240/*λ*_*E*_g__

where *λ*_*E*_g__ (nm) is the absorption edge and *E*_g_ (eV) is the band gap, the band gap of Ti plate, Rb/S/TiO_2_-24, Rb/S/TiO_2_-30, Rb/S/TiO_2_-48, and Rb/S/TiO_2_-72 were calculated to be 3.05 eV, 2.26 eV, 2.25 eV, 2.11 eV, and 2.24 eV, respectively. The absorption intensity of the samples prepared with different hydrothermal times differed from each other and their distinct optical responses were observed in the visible-light region.

**Fig. 6:**
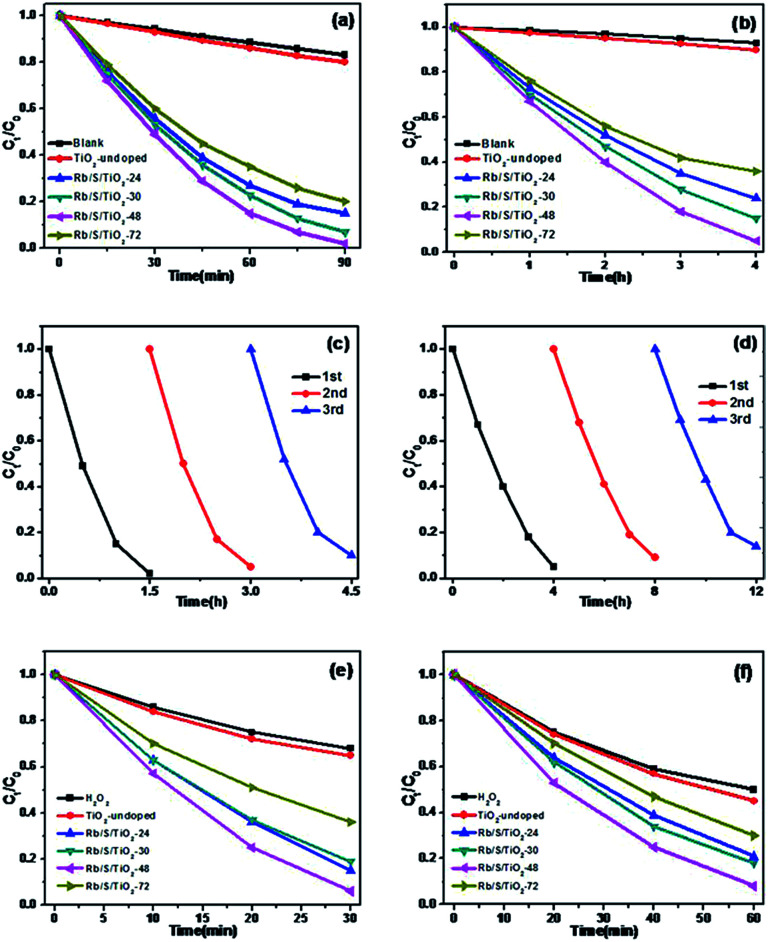
Photocatalytic degradation curves for MO with different samples (MO: 5 mg L^−1^, pH = 7): (a) under UV light, (b) under simulated sunlight, (c, d) recycling of Rb/S/TiO_2_-48 for the removal of MO in UV and simulated sunlight, and (e, f) the synergistic effect of the TiO_2_ film and H_2_O_2_ under UV and simulated sunlight (MO: 5 mg L^−1^, pH = 7, H_2_O_2_: 0.01 mL).

The photocatalytic activities of the TiO_2_ thin films were investigated by performing the degradation reaction of 5 mg L^−1^ methyl orange (MO) using an 80 W high-pressure mercury lamp and a 500 W Xe lamp as the light sources. *C*_0_ is the initial concentration after the adsorption–desorption equilibrium is achieved and *C*_*t*_ is the concentration of MO at time *t*. As shown in Fig. 6a, Rb/S/TiO_2_-48 was able to decolorize 98% of MO under UV light, which is the best activity, while the blank experiment revealed a decomposition ratio of only 17% during the same interval. Moreover, the experiment with TiO_2_-undoped showed a trivial increase in activity by 3% when compared with the blank control.

For simulated sunlight irradiation, Rb/S/TiO_2_-48 also showed the best performance. About 95% of MO was bleached in a 4 h process, which is much higher than that in the reactions with the blank (7%) and TiO_2_-undoped (10%). As shown in Fig. S3,† the PL studies indicate that Rb/S/TiO_2_-48 can effectively inhibit the recombination of the electron–hole pairs, which results in an increase in the photocatalytic activity.^44,45^ Moreover, different photocatalytic activities were observed due to the different thicknesses of the film (Fig. S4†). The film thicknesses of Rb/S/TiO_2_-24, Rb/S/TiO_2_-30, Rb/S/TiO_2_-48, and Rb/S/TiO_2_-72 are 9.65 μm, 14.7 μm, 24.1 μm, and 6.75 μm, respectively. With the increase in the heating time, the thicker oxide film grows directly out from the original surface of the Ti plate. The SEM images show that Rb/S/TiO_2_-48 possessed a large surface area with needle-like structures on the microspheres. However, when the preparation time reached 72 h, the microsphere structures collapsed, leading to a low catalytic efficiency. Rb/S/TiO_2_-72 with highly thick film peeled off during the preparation process because of its fluffy structure, resulting in a poor reproducibility. Hence, all of the abovementioned experimental results indicated that the as-synthesized TiO_2_ films help to promote the degradation of MO. As shown in Fig. S6,† a linear correlation between ln(*C*_*t*_/*C*_0_) and the reaction time (*t*) was obtained, indicating that the decolorization reaction of MO follows the pseudo-first-order kinetics:

2 −ln(*C*_*t*_/*C*_0_) = *kt*

where *C*_*t*_ stands for the real-time concentration of the MO solution, *t* represents the reaction time, and *k* stands for the rate constant. Among all the samples, Rb/S/TiO_2_-48 has the highest rate constants, which are 0.042 min^−1^ in UV light and 0.73 h^−1^ in simulated sunlight.

In addition to the degradation efficiency, the performance reproducibility of the as-synthesized TiO_2_ films is another crucial property to be considered. As shown in Fig. 6c and d, after being used for 3 times, the degradation efficiency of Rb/S/TiO_2_-48 declined by only 8% in UV light and 9% in sunlight, indicating that the TiO_2_ film could be regarded as a stable photocatalyst for the degradation of organic pollutants. The Rb/S/TiO_2_-48 film with moderate and stable structure hardly changed its morphology, hence exhibiting a better performance in the recycling process.

In this study, 0.01 mL H_2_O_2_ was added to the same MO solution with the TiO_2_ film to test the synergistic effect of TiO_2_ film and H_2_O_2_. As shown in Fig. 6e and f, with continuous sampling, TiO_2_-undoped showed a trivial promotion in activity by only 3% (under UV) and 5% (under sunlight) for the oxidation of MO compared to those of the blank experiment. In contrast, Rb/S/TiO_2_-48 still showed the most salient activity with 94% and 92% of MO degraded in the same duration.

Interestingly, the conversion on simultaneously using Rb/S/TiO_2_-48 and H_2_O_2_ is higher than the sum of that from using H_2_O_2_ and Rb/S/TiO_2_-48 individually, which was 81% and 83%, respectively. The synergistic effect of Rb/S/TiO_2_-48 and H_2_O_2_ led to the improvement of up to 13% (under UV) and 9% (under sunlight), indicating a mutual promotion on the degradation of MO between H_2_O_2_ and Rb/S/TiO_2_-48.

**Fig. 7:**
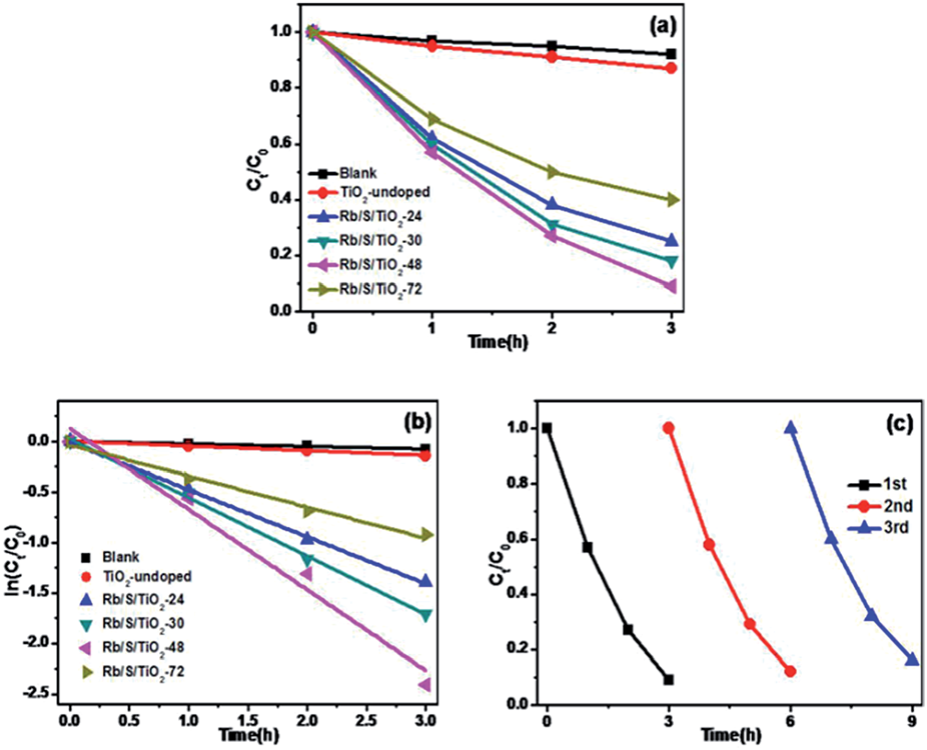
(a) Photocatalytic degradation curves for 4-CP under simulated sunlight (4-CP: 10 ppm, pH = 5.6), (b) kinetic linear simulation curves of the samples, and (c) recycling of Rb/S/TiO_2_-48 in the removal of 4-CP.

In order to prove that the Rb/S/TiO_2_ film is an efficient photocatalyst for a wide range of pollutants, the photocatalytic performance of different samples was evaluated for the degradation of 10 ppm 4-CP solution, which is a type of common colourless pollutants. The light source is the same 500 W Xe lamp and TiO_2_-undoped was also used as the control to degrade the same 4-CP solution. Fig. 7a shows the degradation of 4-CP in the presence of different samples. *C*_0_ and *C*_*t*_ are the initial concentration and real-time concentration of 4-CP, respectively. In a 3 h irradiation process, without any photocatalyst in the solution, only 8% of 4-CP was removed, while in the presence of TiO_2_-undoped, the removal efficiency reached 13%. In contrast, the degradation was clearly enhanced by the as-prepared TiO_2_ films. Among four samples, Rb/S/TiO_2_-48 caused the highest degradation of 91%. As shown in Fig. 7b, all of the degradation data are well-fitted by the pseudo-first-order kinetics. Rb/S/TiO_2_-48 has the highest rate constant (0.8 h^−1^). The rate constants of the other three samples are 0.46 h^−1^ (Rb/S/TiO_2_-24), 0.58 h^−1^ (Rb/S/TiO_2_-30), and 0.3 h^−1^ (Rb/S/TiO_2_-72), which are much higher than that of TiO_2_-undoped (0.046 h^−1^). Because Rb/S/TiO_2_-48 showed the best efficiency among all of the samples, the recycling experiments were carried out with Rb/S/TiO_2_-48 only (Fig. 7c). After being used for three times, the conversion of 4-CP by Rb/S/TiO_2_-48 can still reach 84%, indicating the remarkable stability of this TiO_2_ film.

**Fig. 8:**
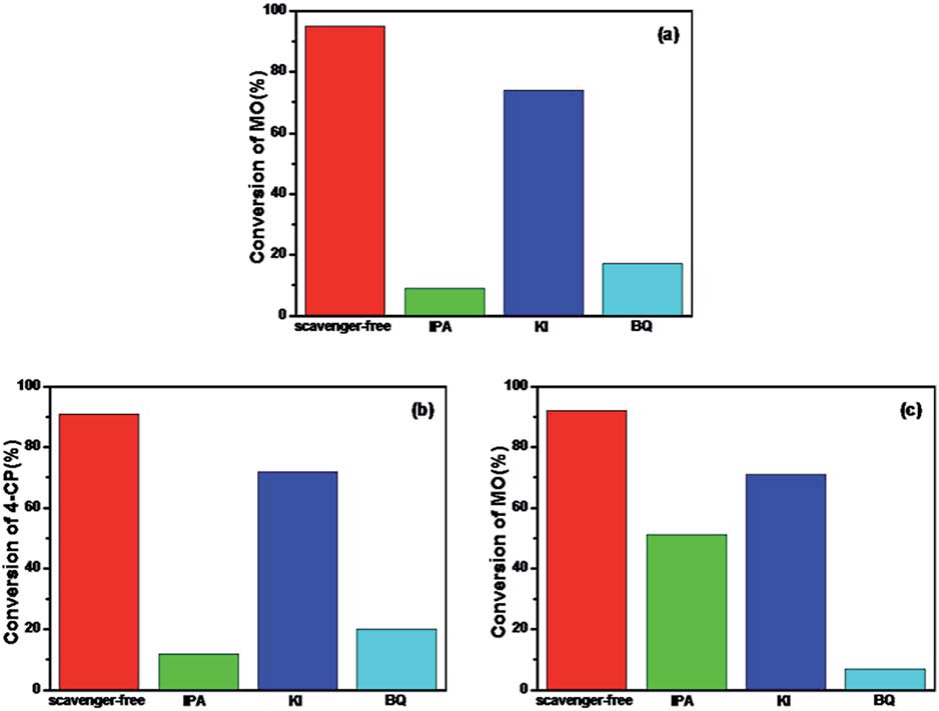
Photocatalytic degradation by Rb/S/TiO_2_-48 in the presence of IPA, KI, and BQ under simulated sunlight: (a) MO for 4 h, (b) 4-CP for 3 h, and (c) MO with the presence of H_2_O_2_ for 1 h.

To further understand the mechanism of the degradation of MO and 4-CP under simulated sunlight, the reactive oxygen species (ROS) and hole scavenging experiment was carried out to identify the active species generated during the process. In this study, isopropanol (IPA), KI, and benzoquinone (BQ) were used as the scavengers to study the effect of hydroxyl radical (˙OH), h_VB_^+^, and superoxide radical (˙O_2_^−^), respectively.^46,47^ As shown in Fig. 8a, for different scavengers, various degrees of decline were observed in the MO degradation. After irradiation for 4 h, the conversion of MO by Rb/S/TiO_2_-48 without scavenger was 95%. Both 1 mM IPA and 1 mM BQ resulted in an evident decrease in the degradation rate. The conversion of MO inhibited by IPA reaches only 9%, which is lower than that in the presence of BQ (17%). With the addition of 1 mM of KI to the Rb/S/TiO_2_-48 and MO system, the conversion reached 69%, which was inhibited by KI by some extent inhibited by KI. From the KI system, it can be concluded that abundant h_VB_^+^ species exist owing to the efficient separation of the e_CB_^−^ and h_VB_^+^.

Similar to the results obtained with the MO solution, in Fig. 8b, the conversion of 4-CP by Rb/S/TiO_2_-48 without the scavenger under simulated sunlight can reach 91%. When IPA or BQ was added to the 4-CP solution, the conversion of 4-CP was only 12% and 20%, respectively. The conversion in the presence of KI for 4-CP was 72%, indicating that the h_VB_^+^ species also contributed to the photocatalysis process. Interestingly, the conversion of MO and 4-CP is almost the same in the IPA and BQ systems. Therefore, it can be concluded that ˙OH and ˙O_2_^−^ are the most active species for the degradation of the contaminants. For the system containing H_2_O_2_ (Fig. 8c), IPA and KI did not show any conspicuous inhibition for the degradation of MO. In contrast, nearly complete inhibition was observed when BQ was added. The conversion of MO was only 7%, suggesting that ˙O_2_^−^ was the key radical for the removal of MO. Interestingly, the reaction rate was not significantly inhibited by KI. The reason may be that the holes are scavenged by H_2_O_2_ adsorbed on the surface of the film rather than by KI.

**Fig. 9:**
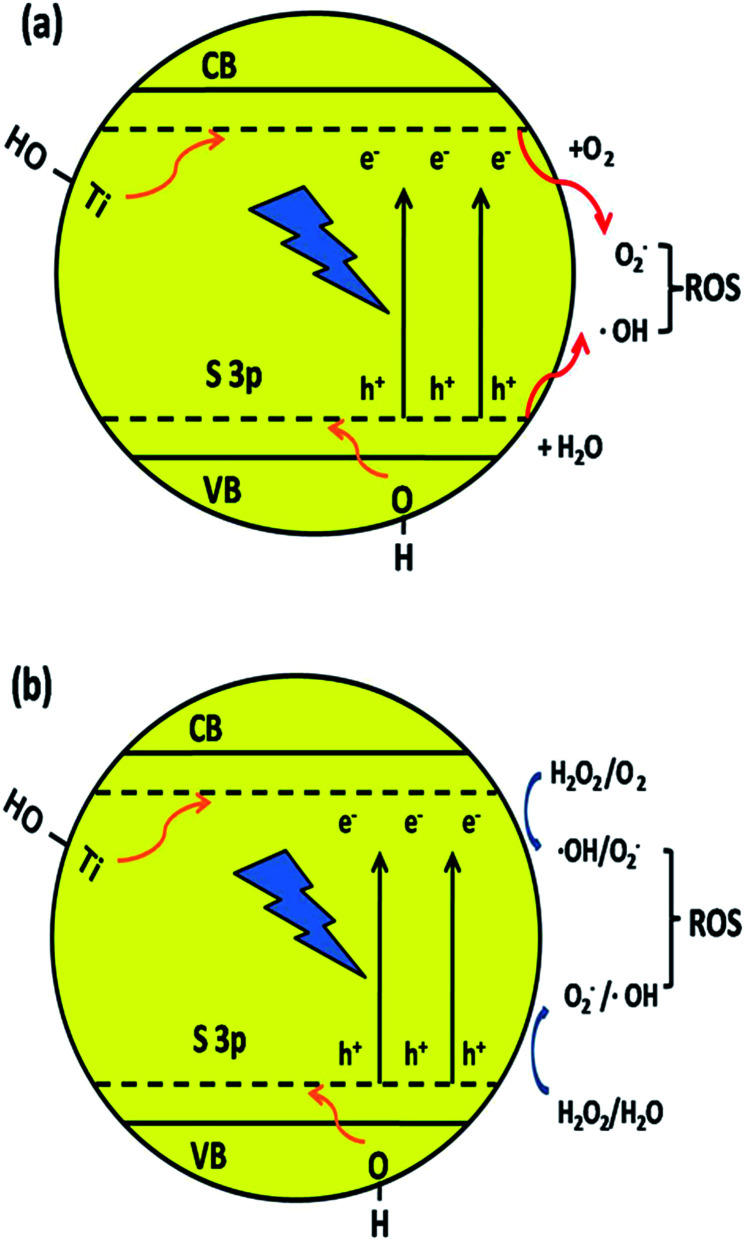
Mechanism of narrowing the band gap of TiO_2_ and the formation of hydroxyl radicals by the synergic effect of element co-doping and surface hydroxyls.

The S element in the material lattice and hydroxyls on the surface of the film could enhance the photocatalytic property by narrowing the band gap of TiO_2_, which is illustrated in Fig. 9a. The –OH group can raise the valence band (VB) and Ti–OH can downshift the conduction band (CB) by inhibiting the electrons coming from neighboring Ti atoms. The S atom is doped into the TiO_2_ lattice and occupies the Ti site, which will also donate electrons to the O atoms to form ˙O_2_^−^ anions. The S^3p^ orbital is at a higher energy than the O^2p^ orbital, which makes the anti-bonding orbital deeper in the band gap and the impurity levels in TiO_2_ also result in a decline in the band gap width. The TiO_2_ film has a better absorption in the visible-light region, which also improves the separating efficiency of the photon-generated carrier. When irradiated, the activated TiO_2_ film generates electrons and holes, which are regarded as the oxidation and reduction agents. Electrons are excited from the valence band (VB) of TiO_2_ to the conduction band (e_cb_^−^), resulting in the formation of electron vacancy (holes) in the valence band (h_vb_^+^). The photo-generated electrons will react with oxygen to form superoxide radical (˙O_2_^−^) and the water molecules can be oxidized by h_vb_^+^ to generate a hydroxyl radical (˙OH). When H_2_O_2_ is added to the system (Fig. 9b), according to Kőrösi *et al.*,^48^ additional ˙OH and ˙O_2_^−^ are formed following eqn (6) and (7). Compared with the system without H_2_O_2_, in the system with H_2_O_2_, the excess radicals increase the oxidation capacity, leading to better degradation efficiency. The mechanism is depicted as follows (eqn (3)–(8)):

3 S/TiO_2_ + *hν* → e_cb_^−^ + h_vb_^+^

4 h_vb_^+^+ H_2_O → H^+^+ ˙OH

5 e_cb_^−^ + O_2_ → ˙O_2_^−^

6 H_2_O_2_ + e_cb_^−^ → ˙OH + OH^−^

7 H_2_O_2_ + h_vb_^+^ + 2OH^−^ → ˙O_2_^−^ + 2H_2_O

8 MO/4-CP + ROS → degradation products

## Conclusions

In this paper, an efficient Rb_2_SO_4_-modified Ti-based TiO_2_ sheet with a thin film was prepared through hydrothermal reaction and calcination, which is highly applicable and repeatable without any harm to the environment. The composite structures were investigated by FT-IR, XRD, SEM, XPS, PL, and UV-vis DRS measurements. All of the photocatalysts were used to degrade the MO and 4-CP solutions. H_2_O_2_ was also added to the reaction system to test the oxidation effect. The results indicated that the as-prepared thin film was effective for the removal of organic pollutants under both UV and simulated sunlight. The best degradation performance was obtained for Rb/S/TiO_2_-48 after calcination. Moreover, when H_2_O_2_ was added to the solution, the efficiency caused by the synergistic effect of both Rb/S/TiO_2_-48 and H_2_O_2_ on degrading MO could reach 13% (under UV) and 9% (under sunlight), which was the most effective among all of the samples. Furthermore, Rb/S/TiO_2_-48 exhibited great stability for cyclic utilization. The materials reported in this paper have exhibited promising potential in organic wastewater treatment.

## Conflicts of interest

There are no conflicts to declare.

## Supplementary Material

RA-008-C7RA12757J-s001
