# Supplementary material for: Hydrothermal in situ synthesis of Rb and S co-doped Ti-based TiO2 sheet with a thin film showing high photocatalytic activities
Source: RSC Adv. 2018 Mar 21;8(20):11247–54. doi: 10.1039/c7ra12757j (PMC9078943; doi:10.1039/c7ra12757j)
Supplement: RA-008-C7RA12757J-s001 [file RA-008-C7RA12757J-s001.pdf]

Electronic Supplementary Material (ESI) for RSC Advances.
This journal is © The Royal Society of Chemistry 2018

Supporting Information:

Hydrothermal in situ synthesis of Rb and S co-doped Ti-based TiO_2 sheet with thin film showing high photocatalytic activities

Yuhan Wang, Chunli Kang, Dongmei Huang, Kunkun Xiao, Ling Zhu, Fang Liu and Tao Tian*

Key Laboratory of Groundwater Resources and Environment, Jilin University, Ministry of Education, Changchun, 130021, Jilin, P. R. China.

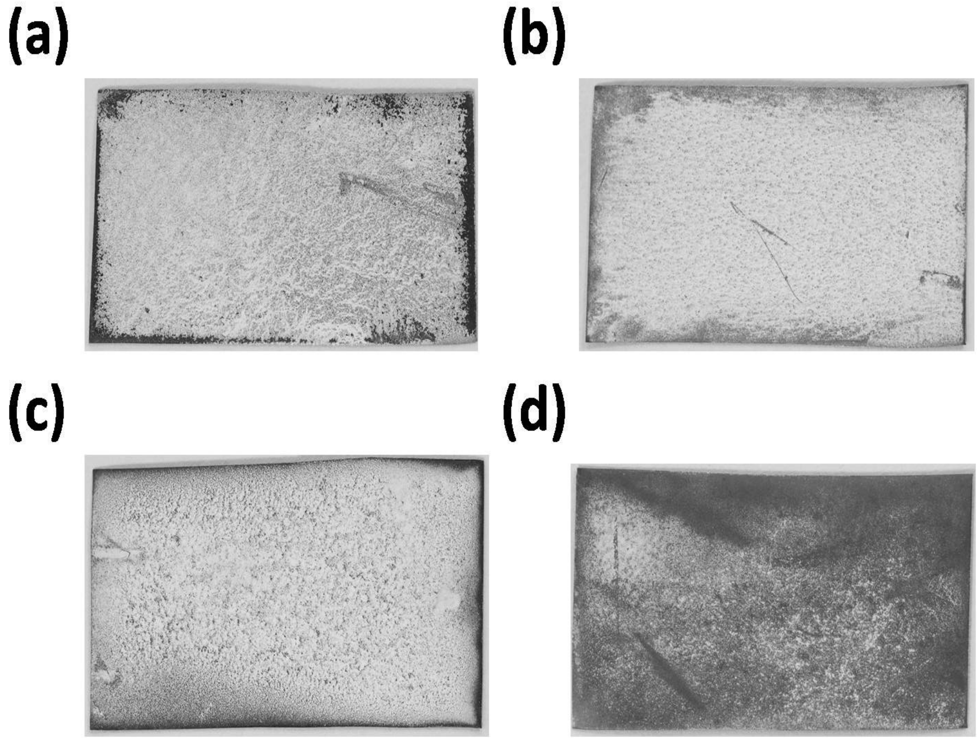

Fig. S1 Images of the Rb_2SO_4 modified film: (a) Rb/S/TiO_2-24, (b) Rb/S/TiO_2-30, (c) Rb/S/TiO_2-48, (d) Rb/S/TiO_2-72

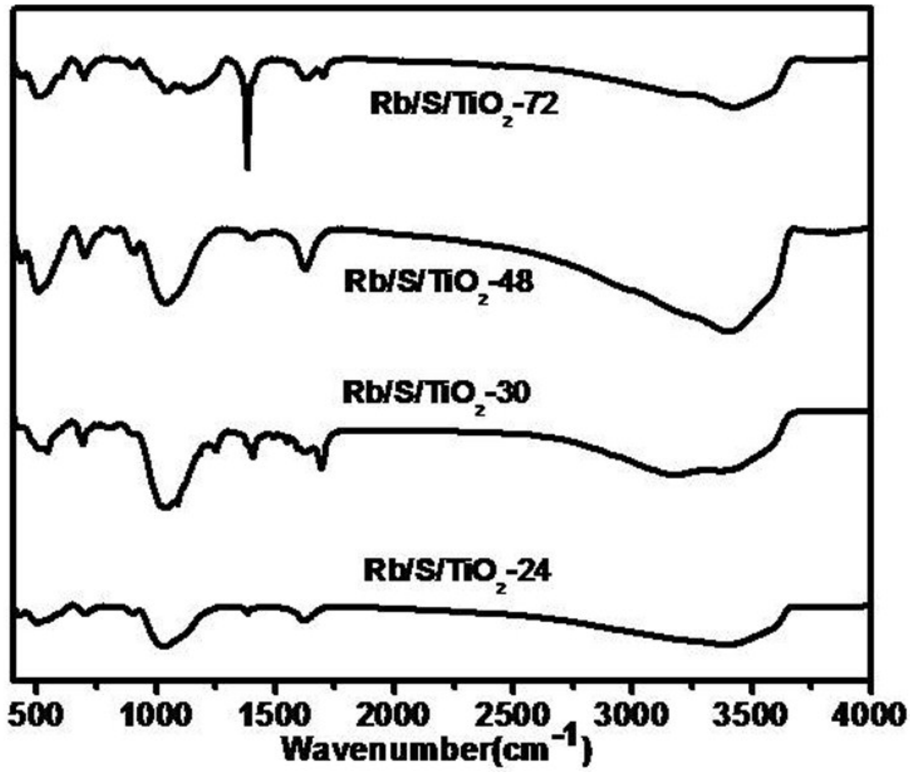

Fig. S2 FT-IR spectra of Rb_2SO_4 modified film.

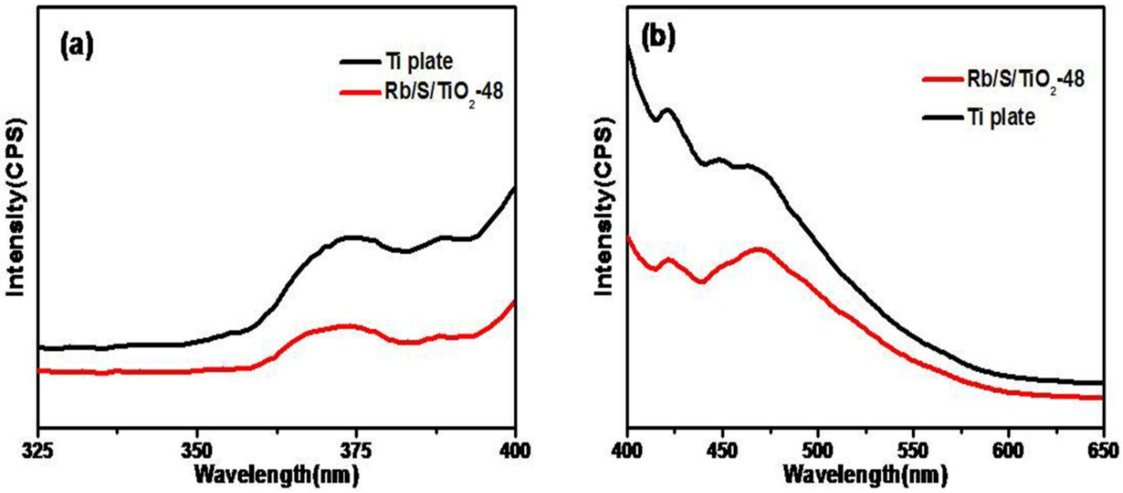

Fig. S3 PL spectra of the samples: (a) Excitation Spectra and (b) Emission Spectra.

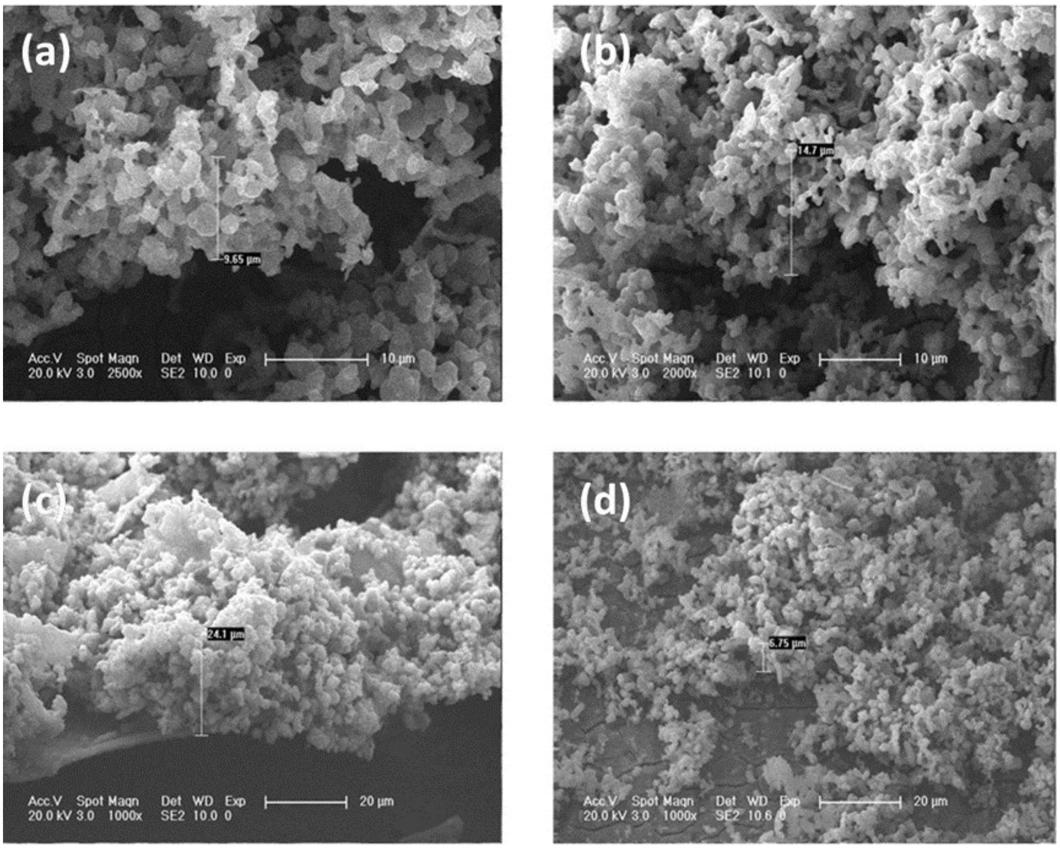

Fig. S4 SEM images of cross section of the film: (a) Rb/S/TiO_2-24, (b) Rb/S/TiO_2-30, (c) Rb/S/TiO_2-48, (d) Rb/S/TiO_2-72.

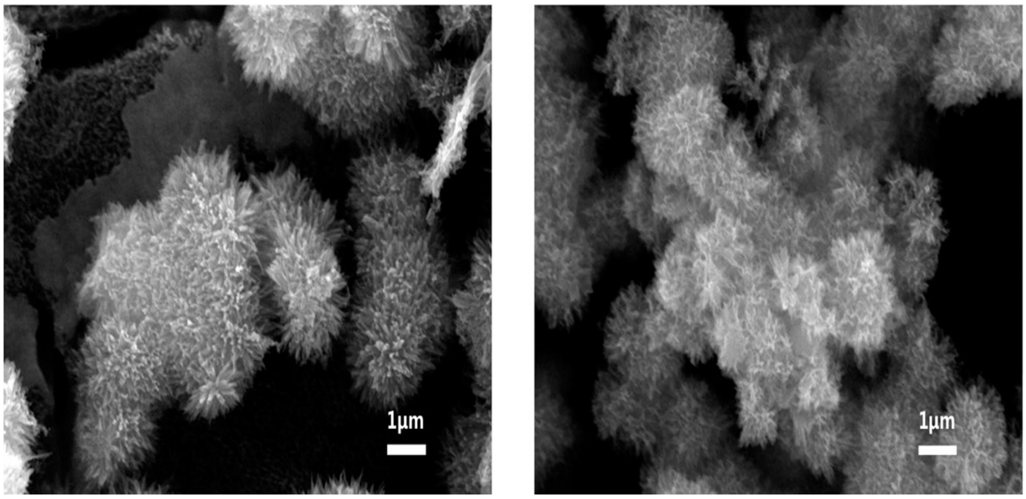

Fig. S5 SEM images of (a) $Rb/S/TiO_2$-24, (b) $Rb/S/TiO_2$-30

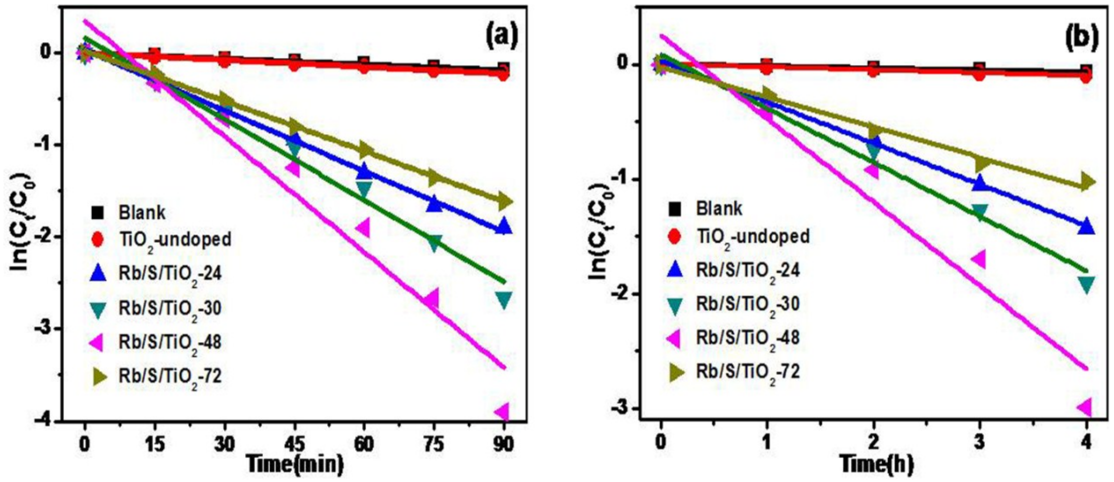

Fig. S6 Kinetic linear simulation curves of samples for the removal of MO: (a) under UV light, (b) under simulated sunlight.

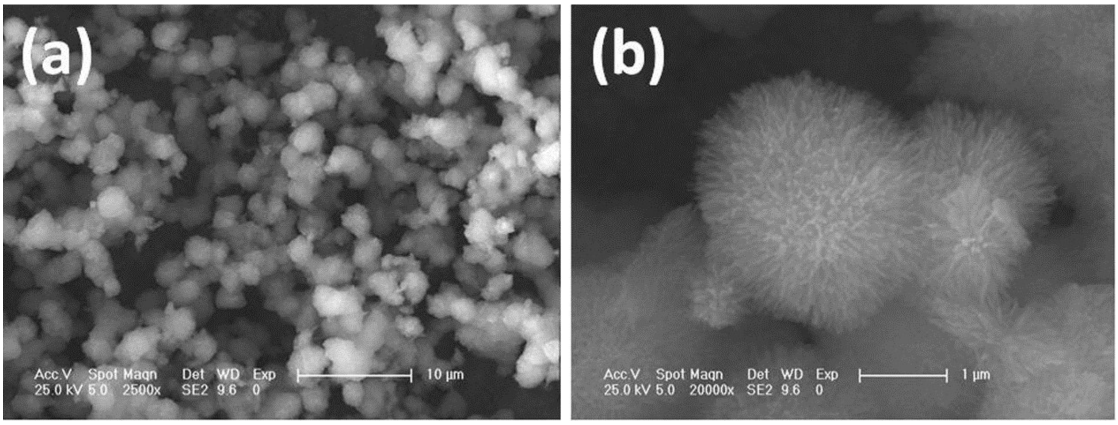

Fig. S7 SEM images of $Rb/S/TiO_2$-48 after being used for 3 times.

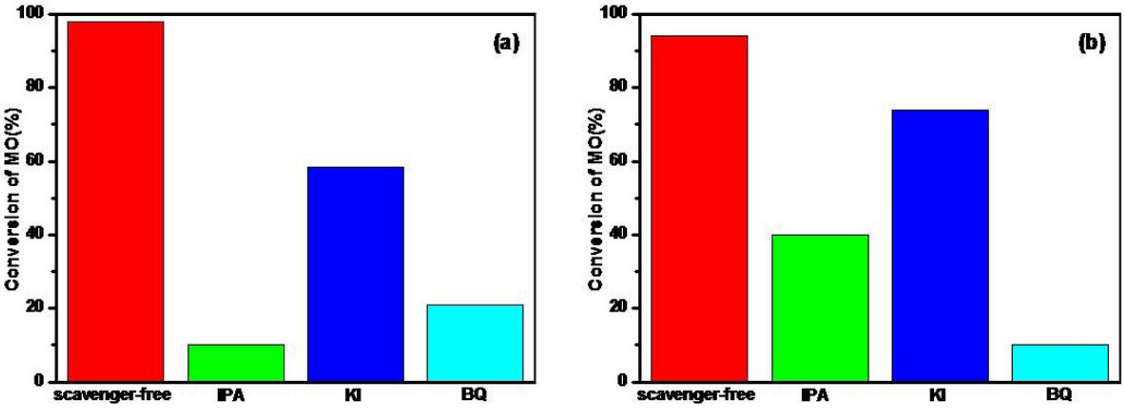

Fig. S8 Photocatalytic degradation by $Rb/S/TiO_2$-48 in the presence of IPA, KI and BQ under UV light: (a) MO for 90min, (b) MO with the presence of H_2O_2 for 30min.

Table S1 Detailed surface elemental composition of Rb/S/TiO_2-24.

Element	State	Relative Content (%)
Ti	Ti 2p	16.7
S	S 2p	3.25
Rb	Rb 3d	3.78
O	O 1s	75.72
N	N 1s	0.15
C	C 1s	0.4

Table S2 Detailed surface elemental composition of Rb/S/TiO_2-30.

Element	State	Relative Content (%)
Ti	Ti 2p	16.46
S	S 2p	3.26
Rb	Rb 3d	3.76
O	O 1s	75.92
N	N 1s	0.2
C	C 1s	0.4

Table S3 Detailed surface elemental composition of Rb/S/TiO_2-48.

Element	State	Relative Content (%)
Ti	Ti 2p	16.63
S	S 2p	3.2
Rb	Rb 3d	3.78
O	O 1s	75.8
N	N 1s	0.2
C	C 1s	0.39

Table S4 Detailed surface elemental composition of Rb/S/TiO_2-72.

Element	State	Relative Content (%)
Ti	Ti 2p	16.65
S	S 2p	3.3
Rb	Rb 3d	3.72
O	O 1s	75.72
N	N 1s	0.2
C	C 1s	0.41

Table S5 Scavengers studies for different reactive species in MO and 4-CP photodegradation.

Target species	Scavenger	Scavenger concentration (mM)
•OH	IPA	1
h^+	KI	1
•O_2^-	BQ	1
